# Granzyme B Contributes to the Optimal Graft-Versus-Tumor Effect Mediated by Conventional CD4^+^ T Cells

**Published:** 2016-04-30

**Authors:** Wei Du, Nicholas D. Leigh, Guanglin Bian, Emad Alqassim, Rachel E. O'Neill, Lin Mei, Jingxin Qiu, Hong Liu, Philip L. McCarthy, Xuefang Cao

**Affiliations:** 1 Department of Immunology, Roswell Park Cancer Institute, Buffalo, USA; 2 Department of Internal Medicine, University at Buffalo, Buffalo, USA; 3 Department of Pathology, Roswell Park Cancer Institute, Buffalo, USA; 4 Department of Medicine, Roswell Park Cancer Institute, Buffalo, USA

**Keywords:** T cells, Allogeneic hematopoietic cell transplantation (allo-HCT), Graft-versus-tumor (GVT) effect, Graft-versus-host-disease (GVHD), Granzyme B (GzmB)

## Abstract

Granzyme B (GzmB) is a key cytotoxic molecule utilized by T cells to kill pathogen-infected cells or transformed tumor cells. Previous studies using allogeneic hematopoietic cell transplantation (allo-HCT) murine models showed that GzmB is required for CD8^+^ T cells to cause graft-versus-host disease (GVHD). However, our recent study demonstrated that GzmB-mediated damage of CD8^+^ T cells diminished their graft-versus-tumor (GVT) activity. In this study, we examined the role of GzmB in GVT effect mediated by conventional CD4^+^CD25^−^ T cells (CD4^+^ Tcon). GzmB^−/−^CD4^+^ Tcon cells exhibited decreased GVT activity compared to wild-type (WT) CD4^+^ Tcon cells, suggesting that GzmB is required for the optimal GVT activity of CD4^+^ Tcon cells. On the other hand, GzmB^−/−^ CD4^+^CD25^+^ regulatory T cells were as suppressive as WT regulatory T cells in suppressing GVT activity, which is consistent with our previous report showing that GzmB is not required for regulatory T cell-mediated suppression of GVHD. These results demonstrate that GzmB causes opposite impacts on GVT effect mediated by CD4^+^CD25^−^ versus CD8^+^ T cells. Interestingly, GzmB^−/−^ total T cells exhibited GVT activity equivalent to that of WT total T cells, suggesting that the opposite impacts of GzmB on the GVT effect of CD4^+^CD25^−^ versus CD8^+^ T cells may neutralize each other, which can only be observed when an individual T cell subset is examined. Importantly, these differential roles suggest that targeting GzmB in selective T cell subsets may have the potential to enhance the beneficial GVT effect.

## INTRODUCTION

Allogeneic hematopoietic stem cell transplantation (allo-HCT) is a potentially curative treatment for leukemia, lymphoma, other hematologic malignancies and immunological diseases [1]. It has been recognized that donor-derived immune cells can identify and attack tumor cells in the host, producing a unique and beneficial immune response that was defined as the graft-versus-tumor (GVT) effect [2,3]. However, graft-versus-host-disease (GVHD), which develops from the attack of host normal tissues by donor allogeneic lymphocytes, limits the success of allo-HCT [2,3]. To date, most of the strategies to control GVHD are broadly immunosuppressive, but not always successful and may lead to other adverse effects such as infection or cancer relapse [4,5]. Therefore, it remains a major challenge to identify novel methods that can separate GVHD from the desired GVT effect.

Several major T cell populations have been shown to be involved in GVHD and GVT effect. While CD8^+^ and CD4^+^CD25^−^ conventional T cells are known to mediate GVHD and GVT activity, CD4^+^CD25^+^ regulatory T cells may suppress GVHD and GVT effect [5]. At the level of effector molecules, three major pathways have been described for allogeneic T cell-mediated cytotoxicity: Fas/Fas ligand (FasL), perforin/granzymes and cytotoxic cytokine pathways [6–9]. Perforin is known to deliver granzymes into target cells, where granzymes induce apoptosis by cleaving critical intracellular substrates [8,9]. Among these cytotoxic molecules, granzyme B (GzmB) was shown to be required in CD8^+^ T cell-mediated GVHD [10,11]. Interestingly, while our recent study confirmed that host mice receiving GzmB^−/−^ CD8^+^ T cells had decreased severity of GVHD compared to mice receiving WT CD8^+^ T cells, we found that GzmB^−/−^CD8^+^ T cells exhibited GVT activity that was significantly higher than that of WT CD8^+^ T cells [12]. This finding suggests that GzmB causes dual detriment for CD8^+^ T cells after allo-HCT in that it not only causes GVHD but also impairs the desired GVT effect. This unexpected finding with CD8^+^ T cells prompts a new hypothesis that GzmB may have different functions in various T cell populations in the setting of allo-HCT. Based on this hypothesis, we have performed this study to examine the role of GzmB in GVT effect mediated by CD4^+^CD25^−^ conventional T cells and CD4^+^CD25^+^ regulatory T cells. In this work, we provide new evidence to show that GzmB contributes to the optimal GVT activity of conventional CD4^+^CD25^−^ T cells, which is opposite to its negative impact on the GVT activity of CD8^+^ T cells. In addition, we show that GzmB is not involved in CD4^+^CD25^+^ regulatory T cell-mediated suppression of GVT effect, which is consistent with our previous report showing that GzmB is not required in CD4^+^CD25^+^ regulatory T cell-mediated suppression of GVHD [13]. These differential roles suggest that targeting GzmB in selected T cell subsets may provide a promising strategy for separating GVHD from the beneficial GVT effect.

## MATERIALS AND METHODS

### Animals and Tumor cells

C57BL/6 (H-2^b^) and 129/SvJ (H-2^b^) WT mice were obtained from the Jackson Laboratory. BALB/c (H-2^d^) mice were purchased from NCI and Charles River Laboratory. GzmB^−/−^ mice in the C57BL/6 strain and 129/SvJ strain were developed and maintained as previously described [12,14,15]. A20 lymphoma cells, derived from BALB/c strain, were transduced to express luciferase as previously described and used for bioluminescence imaging to measure tumor burden in vivo [12,14,16,17]. All mice were maintained in SPF housing, and all experiments were conducted in accordance with the animal care guidelines at Roswell Park Cancer Institute, using protocols approved by animal studies committee.

### Reagents and Antibodies

Pan T isolation kits, regulatory T cell isolation kits, and anti-CD90.2 microbeads were purchased from Miltenyi Biotec. Antibodies including anti-mouse CD3, TCRβ, CD4, CD8, CD25, H-2K^b^, H-2K^d^, and CD16/32 were purchased from eBioscience.

### Donor cell preparation

The donor mice were in the C57BL/6J strain or the 129/SvJ strain (both H-2^b^). All donor bone marrow (BM) cells were isolated from WT mice. T cell depletion (TCD) was performed with auto-MACS by using anti-CD90.2 microbeads (Miltenyi Biotec). Donor CD4^+^CD25^−^ T cells (purity > 90%) were purified from the spleens by using Pan T isolation kit II combined with biotin-conjugated anti-CD8 and anti-CD25 antibodies. Donor CD4^+^CD25^+^ regulatory T cells (purity > 90%) were purified from the spleens by using mouse regulatory T cell isolation kit (Miltenyi Biotec). Donor CD8^+^ T cells (purity > 90%) were purified from the spleens by using Pan T isolation kit II combined with biotin-conjugated anti-CD4 antibody. Donor total T cells (purity > 90%) were purified from the spleens by using Pan T isolation kit II.

### GVT model

The BALB/c (H-2^d^) host mice were irradiated with 8 Gy. One day later, the host mice were inoculated through lateral tail vein with 1–100×10^4^ luciferase-expression A20 lymphoma cells. Immediately afterwards, via another lateral tail vein injection, the mice received 2×10^6^ TCD-BM cells only or combined with 1–10×10^4^ CD4^+^CD25^−^ T cells or 4×10^5^ CD8^+^ T cells isolated from WT or GzmB^−/−^ mice. For the in vivo regulatory T cell suppression experiments, 8–9×10^4^ WT or GzmB^−/−^ CD4^+^CD25^+^ regulatory T cells isolated from C57BL/6 donor mice were mixed with CD4^+^CD25^−^ or CD8^+^ T cells before injection. Bioluminescence imaging was performed to monitor tumor burden as described [14,18]. Tumor burden was expressed as photon flux (photons/sec).

## RESULTS

### GzmB contributes to the optimal GVT activity of conventional CD4^+^CD25^−^ T cells

**Figure 1:**
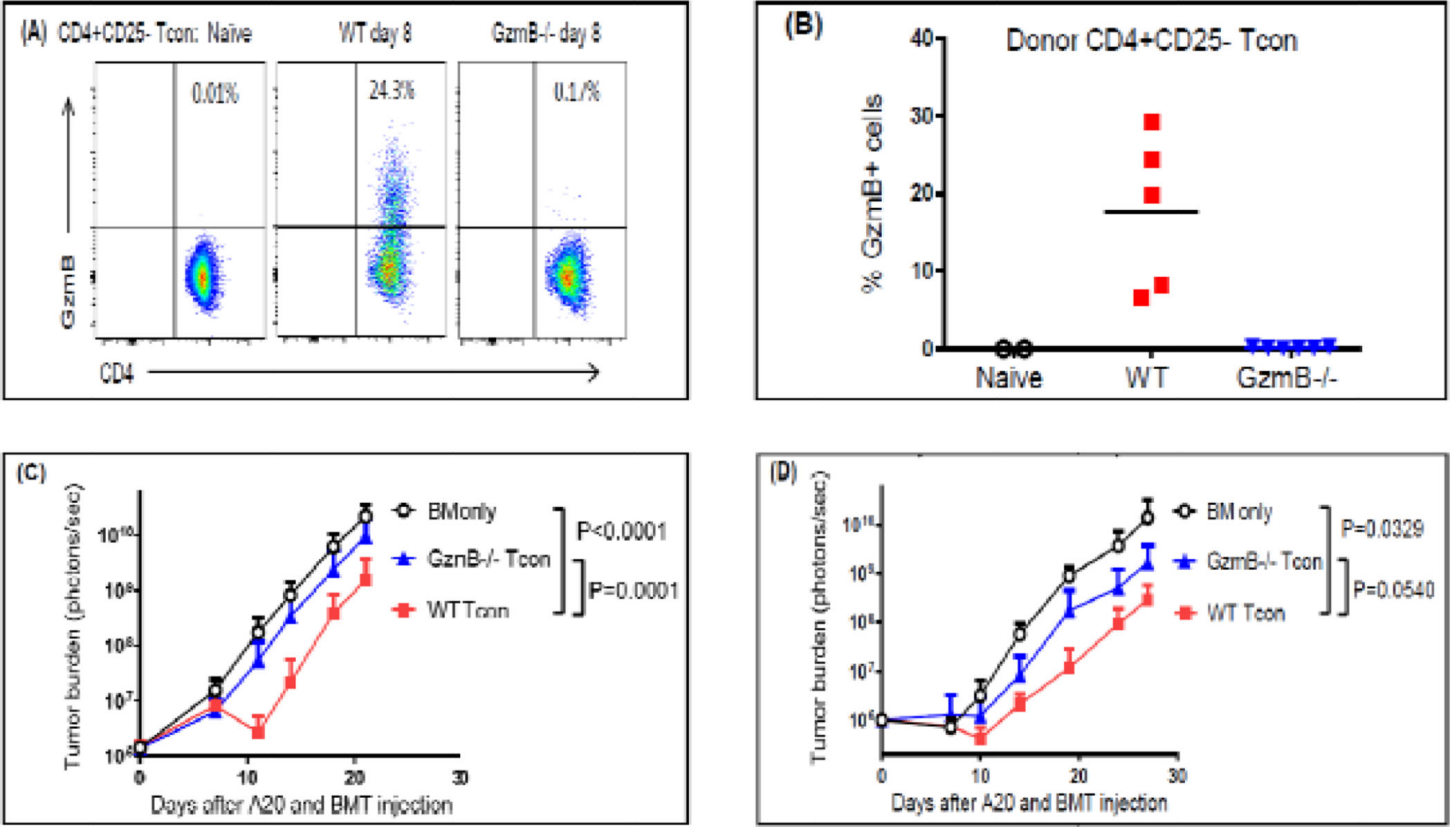
CD4^+^CD25^−^ T cell-mediated GVT effect is diminished in the absence of GzmB **(A)** BALB/c (H-2d) host mice were irradiated with 800 rad at day −1. On day 0, host mice were injected with 2×106 TCD-BM cells alone or combined with 2×104 WT or GzmB−/− CD4+CD25− Tcon cells purified from C57BL/6 (H-2b) donor mice. On day 8, total cells harvested from the host spleens were analyzed with flow cytometry to examine GzmB expression in donor-derived T cells. Purified pre-HCT naive CD4+CD25− T cells were also examined as controls. Representative dot plots are gated on donor-derived T cells (H-2Kb+CD3+CD4+). **(B)** Summary data of the percentages of donor CD4+CD25− Tcon cells described in (A) that are GzmB+ are shown with each point representing an individual mouse. **(C)** BALB/c (H-2d) host mice were irradiated with 800 rad at day −1. On Day 0, mice were inoculated IV with 1×10^5^ luciferase-expressing A20 tumor cells and subsequently injected with 2×10^6^ TCD-BM cells alone or combined with 2×10^4^ WT or *GzmB^−/−^* CD4^+^CD25^−^ Tcon cells isolated from C57BL/6 (H-2b) donor mice. **(D)** BALB/c (H-2d) host mice were irradiated with 800 rad at day −1. On Day 0, mice were inoculated IV with 1×10^4^ or luciferase-expressing A20 tumor cells and subsequently injected with 2×10^6^ TCD-BM cells alone or combined with 1×10^4^ WT or *GzmB^−/−^* CD4^+^CD25^−^ Tcon cells isolated from C57BL/6 (H-2b) donor mice. Tumor burden was measured with bioluminescence imaging and shown as mean ± SD. 5–10 host mice were used in each group for each experiment. Two-way ANOVA was performed for statistical analyses.

To study the function of GzmB in conventional CD4^+^CD25^−^ T (Tcon) cells in the setting of allo-HCT, we first examine GzmB expression in these cells. While GzmB protein is virtually non-detectable in naïve CD4^+^CD25^−^ Tcon cells, substantial GzmB expression is detected in donor-derived CD4^+^CD25^−^ Tcon cells harvested from the spleens of the host mice on day 8 after allo-HCT (Figure 1A–1B). To examine the role of GzmB in GVT effect mediated by CD4^+^CD25^−^ Tcon cells, we utilized A20 tumor cells, which are B cell lymphoma cells derived from BALB/c strain and express normal level of MHC class II. We have performed two independent experiments using a high dose (1×10^5^ in Figure 1C) and a low dose (1×10^4^ in Figure 1D) of luciferase-expressing A20 cells respectively to inoculate the BALB/c host mice. Immediately after tumor inoculation, the host mice were transplanted with 2×10^6^ TCD-BM cells alone or combined with WT or *GzmB^−/−^* CD4^+^CD25^−^ Tcon cells isolated from C57BL/6 (H-2^b^) donor mice. In a separate GVHD study, we have found that CD4^+^CD25^−^ Tcon cells are very potent in inducing lethal acute GVHD in that 2–5×10^4^ B6 Tcon cells would cause substantial lethality to BALB/c hosts within 10 days after allo-HCT. Therefore, we used low doses of (1–2×10^4^) Tcon cells in these GVT experiments to assure that the majority of the host mice survive long enough for us to measure GzmB-dependent GVT activity. Specifically, 2×10^4^ CD4^+^CD25^−^ Tcon cells were used for the host group with high tumor dose and 1×10^4^ CD4^+^CD25^−^ Tcon cells were used for the host group with low tumor dose. Using bioluminescence imaging to measure tumor burden, we have observed similar results with these two tumor doses showing that GzmB^−/−^ CD4^+^CD25^−^ Tcon cells are less effective than WT CD4^+^CD25^−^ Tcon cells in controlling tumor growth (Figure 1C–1D). These results demonstrate that GzmB deficiency reduced the GVT activity of CD4^+^CD25^−^ Tcon cells, suggesting that GzmB contributes to the optimal GVT effect mediated by CD4^+^CD25^−^ Tcon cells.

### GzmB is not required for regulatory T cell-mediated suppression of GVT effect mediated by either CD4^+^CD25^−^ Tcon cells or CD8^+^ cytotoxic T cells

**Figure 2:**
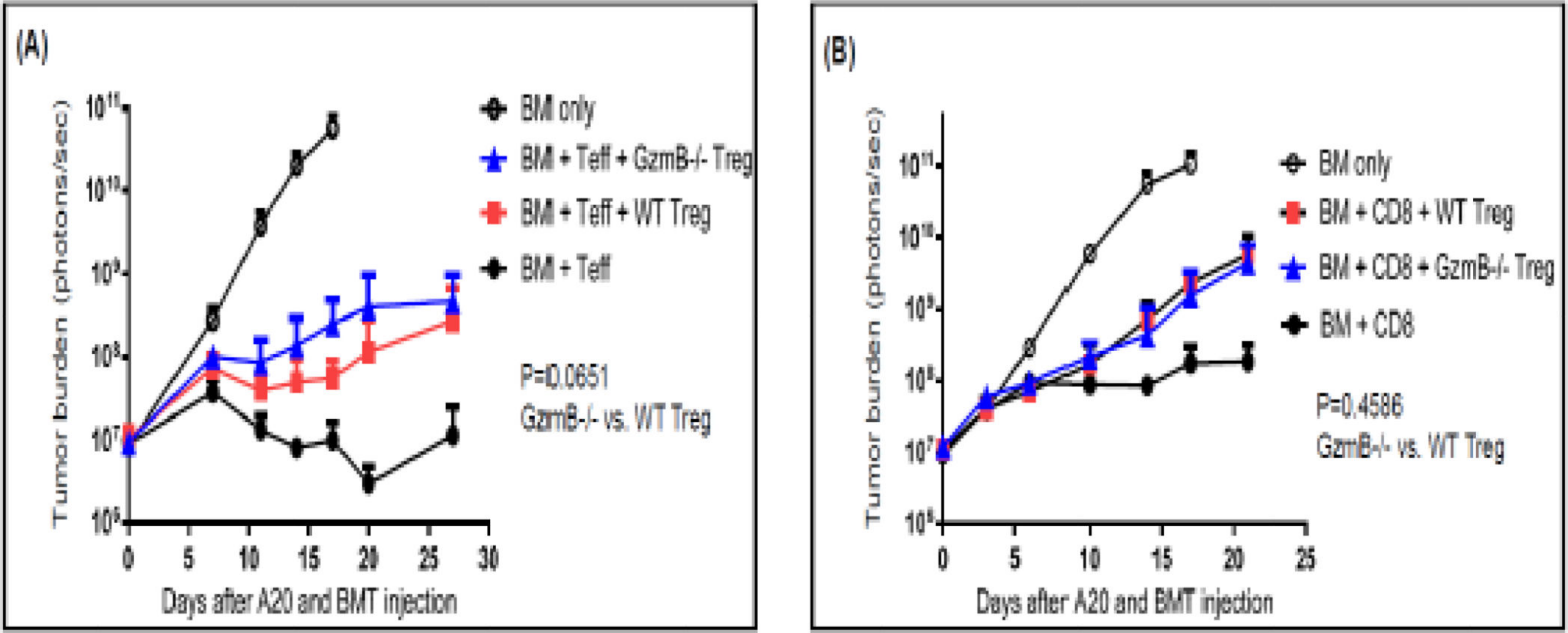
GzmB deficiency did not alter the ability of CD4^+^CD25^+^ Treg cells to suppress GVT effect mediated by CD8^+^ and CD4^+^CD25^−^ T cells **(A)** BALB/c (H-2d) mice were irradiated with 800 rad at day −1. At Day 0, mice were inoculated IV with 1×10^6^ luciferase-expressing A20 cells and subsequently given 2×10^6^ TCD-BM cells alone or combined with 1×10^5^ WT CD4^+^CD25^−^ T cells only or mixed with 8×10^4^ WT or GzmB^−/−^ CD4^+^CD25^+^ Treg cells isolated from C57BL/6 (H-2b) donor mice. **(B)** BALB/c (H-2d) mice were irradiated with 800 rad at day −1. At Day 0, mice were inoculated IV with 1×10^6^ luciferase-expressing A20 cells and subsequently given 2×10^6^ TCD-BM cells alone or combined with 4×105 GzmB^−/−^ CD8^+^ T cells only or mixed with 9×10^4^ WT or GzmB^−/−^ CD4^+^CD25^+^ Tregs isolated from C57BL/6 (H-2^b^) donor mice. Tumor burden was measured with bioluminescence imaging and shown as mean ± SD. 5–10 host mice were used in each group for each experiment. Two-way ANOVA was performed for statistical analyses.

Our previous report with syngeneic tumor models indicated that GzmB is critical for the ability of CD4^+^CD25^+^ regulatory T (Treg) cells to suppress antitumor immune responses mediated by CD8^+^ cytotoxic T cells and natural killer (NK) cells [14]. However, our later studies with allo-HCT models revealed that GzmB is not required for donor Treg cell-mediated suppression of GVHD [13]. These reports left behind an important question regarding whether GzmB is involved in Treg cell-mediated suppression of antitumor immune response in the setting of allo-HCT. To answer this question, we isolated CD4^+^CD25^+^ Treg cells from WT and GzmB^−/−^ donor mice and compared their suppressive activity on GVT effect mediated by either CD4^+^CD25^−^ Tcon cells or CD8^+^ cytotoxic T cells. To assure that Treg-mediated suppression was measurable, we used a high dose of (1×10^5^) CD4^+^CD25^−^ Tcon cells to induce strong GVT activity upon which Treg cells could be tested for hypothetically GzmB-dependent function. At this high dose of CD4^+^CD25^−^ Tcon cells, 5 out of 10 mice receiving only Tcon cells died from GVHD between days 7 and 14 after HCT, while all the mice receiving Tcon combined with Treg cells survived over a months after HCT. As shown in Figure 2, when CD4^+^CD25^+^ Treg cells were added to HCT graft, these Treg cells were able to significantly suppress GVT activity mediated by either CD4^+^CD25^−^ Tcon cells or CD8^+^ T cells. However, GzmB deficiency did not make any significant difference on the suppressive activity of donor Treg cells (Figure 2A–2B.). Together, these results indicate that GzmB is not involved in Treg cell-mediated suppression of GVT effect, which is consistent with our previous report showing that GzmB is not required for donor Treg cell-mediated suppression of GVHD in the allo-HCT models [13].

### GzmB^−/−^ total T cells exhibit equivalent GVT activity to that of WT total T cells

Our recent report showed that GzmB^−/−^ CD8^+^ T cells exhibited significantly enhanced GVT activity compared to WT CD8^+^ T cells, probably due to GzmB-mediated cell autonomous damage of donor CD8^+^ T cells [12]. Intriguingly, new data in this study shows that GzmB^−/−^CD4^+^CD25^−^ Tcon cells exhibit reduced GVT activity compared to WT CD4^+^CD25^−^ Tcon cells.

**Figure 3:**
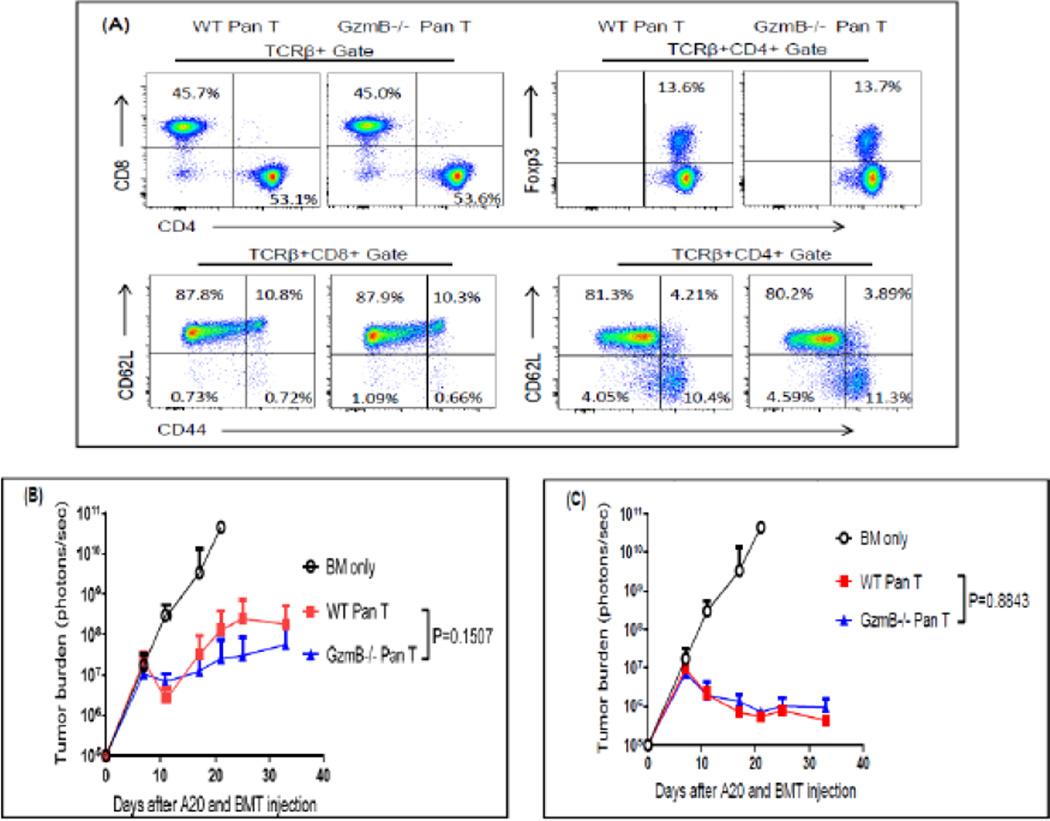
GzmB^−/−^ total T cells exhibited equivalent GVT activity to that of WT total T cells **(A)** Before allo-HCT, Pan T cells isolated from C57BL/6 WT or *GzmB^−/−^* donor mice were examined by flow cytometry for composition of CD8^+^, CD4^+^, CD4^+^Foxp3^+^ subpopulations and phenotypic differentiation via CD44 and CD62L expression. **(B)** BALB/c (H-2^d^) mice were irradiated with 800 rad at day −1. At Day 0, mice were inoculated IV with 1×10^5^ luciferase-expressing A20 cells and subsequently given 2×10^6^ TCD-BM cells alone or combined with 5×10^4^ WT or *GzmB^−/−^* Pan T cells isolated from 129/SvJ (H-2^b^) donor mice. **(C)** BALB/c (H-2^d^) mice were irradiated with 800 rad at day −1. At Day 0, mice were inoculated IV with 1×10^5^ luciferase-expressing A20 cells and subsequently given 2×10^6^ TCD-BM cells alone or combined with 10×10^4^ WT or *GzmB^−/−^* Pan T cells isolated from 129/SvJ (H-2^b^) donor mice. Tumor burden was measured with bioluminescence imaging and shown as mean ± SD. 5–10 host mice were used in each group for each experiment. Two-way ANOVA was performed for statistical analyses.

Put together, these results suggest that GzmB causes opposite impacts on GVT effect mediated by CD4^+^CD25^−^ T cells versus CD8^+^ T cells. However, it is important to note that in the clinical setting where T cell replete allo-HCT is performed, all subsets of T cells are included. Therefore, it becomes an interesting and clinically relevant question whether and how GzmB impacts GVT effect when unfractionated total T cells are used for allo-HCT. To answer this question, we isolated Pan T cells that contain both CD4^+^ (both Tcon and Treg) and CD8^+^ T cells from GzmB^−/−^ and WT donor mice. Importantly, pre-HCT analyses showed that GzmB deficiency does not alter Pan T cells regarding composition of CD8+, CD4+, CD4+Foxp3+ populations or phenotypic differentiation via CD44 and CD62L expression (Figure 3A). To accurately compare the GVT activity of GzmB^−/−^ and WT total T cells, we transplanted a low dose (5×10^4^, Figure 3B) and a high dose (10×10^4^, Figure 3C) of Pan T cells for allo-HCT. As shown in Figure 3B–3C, donor Pan T cells mediated GVT effect in a dose-dependent fashion. However, at both doses, GzmB^−/−^ Pan T cells exhibited GVT activity equivalent to that of WT Pan T cells. Altogether, these results suggest that the opposite impacts of GzmB on the GVT effect of CD4^+^CD25^−^ versus CD8^+^ T cells may neutralize each other

## DISCUSSION

GzmB has been known as a key cytotoxic molecule used by cytotoxic lymphocytes to kill host cells infected by intracellular pathogens or transformed tumor cells. GzmB-mediated killing of target cells involves activation of mitochondria-mediated apoptosis [19,20]. In addition, GzmB can also directly activate caspase-dependent cell death cascade by cleaving the effector caspase 3 and the initiator caspase 8 [19,20]. To date, the importance of GzmB has been implicated in autoimmune disease, infection immunity, tumor immunity, GVHD and GVT effect [19,20]. Regarding different T cell populations, it was initially thought that Fas/FasL system plays a more important role in CD4^+^ T cell-mediated cytotoxic effect, while perforin and GzmB are more restricted to CD8^+^ T cell-mediated cytotoxicity [10,11]. However, recent studies have revealed that there are more complex functions performed by GzmB in various lymphocyte subsets. As shown in our recent report, GzmB is indispensable in CD8^+^ T cell-mediated GVHD while diminishing CD8^+^ T cell-mediated GVT effect [12].

Moreover, in this work focusing on CD4^+^CD25^−^ Tcon cell-mediated GVT effect, we have found that GzmB plays an opposite role to that in CD8^+^ T cells in that GzmB deficiency in CD4^+^CD25^−^ Tcon cells actually diminished GVT effect. Although the underlying mechanism for the opposite roles is not yet completely determined, it may be due to the fundamental difference between how CD4^+^ versus CD8^+^ T cells recognize and interact with their target cells. MHC class I and II molecules are essential for the activation of CD8^+^ and CD4^+^ T cells respectively. GzmB-mediated killing requires direct contact between T cells and their target cells. Almost all tissue cells express MHC class I and therefore all types of cells are susceptible to contact-dependent, GzmB-mediated killing by CD8^+^ T cells. In contrast, most tissue cells donot express MHC class II and consequently are less likely subjected to GzmB-mediated killing by CD4^+^ T cells. In this case, CD4^+^ T cell-mediated damage may mainly work through contact-independent mechanisms, likely via proinflammatory cytokines. However, certain immune cell-derived tumor cells such as A20 cells express MHC class II. It is therefore possible that with MHC class II-mediated recognition and interaction, CD4^+^ T cells can use GzmB to directly kill these target cells and hence meet the definition of CD4^+^ cytotoxic T cells. Accordingly, this potential mechanism may explain why the absence of GzmB in CD4^+^CD25^−^ T cells reduced their GVT activity. On the other hand, this study provides new evidence showing that CD4^+^CD25^+^ Treg cell-mediated suppression of GVT effect works via GzmB-independent mechanisms. This finding further proves that GzmB plays different roles in different T cell subsets. Furthermore, our findings of the differential roles of GzmB in Treg cell-mediated suppression of syngeneic versus allogeneic immune responses suggest that the functional mechanisms of Treg cells are highly dependent on the immune context and the model system.

In conclusion, we believe that the differential roles of GzmB in different T cell populations may open up a new avenue to separate GVHD from the desired GVT effect. For example, CD8^+^ and CD4^+^CD25^−^ T cells can be purified separately and manipulated differently before allo-HCT. Based on our findings, we hope that genetically or pharmacologically inhibiting GzmB function in donor CD8^+^ T cells may result in favorable outcomes. However, the integrity of GzmB function in CD4^+^ T cells may need to be maintained in order to optimize the GVT effect for certain types of blood cancers that are sensitive to GzmB-mediated killing by CD4^+^ T cells.
